# Fatigue Analysis of CFRP-Reinforced Concrete Ribbed Girder Bridge Deck Slabs

**DOI:** 10.3390/polym14183814

**Published:** 2022-09-12

**Authors:** Shuai Tian, Xiaotao Zhang, Wenjing Hu

**Affiliations:** School of Civil Engineering, Liaoning University of Science and Technology, Anshan 114051, China

**Keywords:** bridge engineering, ribbed girder bridges, bridge deck slabs, fatigue damage, strengthening methods, CFRP-reinforced concrete

## Abstract

This study aims to improve the operational safety of reinforced concrete-ribbed beam bridge decks and prolong their service life by performing fatigue analysis of deck slabs reinforced with carbon-fiber-reinforced polymers (CFRP) and other materials. Based on a 16-m-span ribbed girder bridge, five test beams were designed: three reinforced (with CFRP cloth, CFRP mesh, and strip steel plates) and the remaining unreinforced. To simulate the real force of the bridge deck slabs, a PLS-500 electro-hydraulic servo dynamic and static test system was used and static load failure (monotonic graded loading) and fixed-point constant-amplitude fatigue loading tests (fatigue load of 0.515, loading frequency of 5 Hz) were performed. The main fatigue crack appeared when the number of load cycles exceeded 90% of the fatigue life. In the middle of fatigue, the reinforcement material can reduce the deterioration value of the bridge deck by approximately 50%. When it is reinforced at the cumulative damage degree of 0.4, its fatigue life extends by approximately 53.3–78.9%. The fatigue life of the bridge deck slabs reinforced with CFRP cloth or mesh was 22.1–25.6% more than that of those reinforced with strip steel plates. CFRP cloth is best suited for the reinforcement of bridge deck slabs.

## 1. Introduction

Reinforced concrete ribbed girder bridge deck slabs are subjected to material deterioration and fatigue under the repeated activity of large vehicles. The load and fatigue resistance of the bridge deck slabs gradually decrease. To resolve the low performance problems caused by material deterioration and fatigue of reinforced concrete ribbed girder bridge deck slabs, reasonable and effective strengthening of bridge deck slabs has gradually attracted the attention of scientists and technicians. Rahman et al. [1] selected a CFRP grid for the reinforcement of bridge deck slabs and carried out fatigue performance tests in the laboratory. Kongguo et al. [2] selected CFRP sheets for strengthening the deck slab of a solid bridge and analyzed the strengthening effect. Christina et al. [3] carried out comparative tests by selecting different types of mounted bars for bridge deck slabs. Yi et al. [4] analyzed the maintenance and reinforcement technology of a highway bridge deck and reinforcement layer. Li [5] selected FRP for reinforcing reinforced concrete one-way slabs and carried out bending tests. Biliang [6] selected CFRP for strengthening the bridge deck slab and carried out ductility tests. Chanhao [7] selected CFRP sheets for the reinforcement of bridge deck slabs and evaluated the fatigue resistance. Fang [8] selected BFRP grids for strengthening the bridge deck slab and carried out bending tests, while Yu et al. [9] selected FRP bars reinforcement for bridge deck slabs and carried out bending tests.

Damaged concrete bridge deck slabs can be reinforced with various materials used for damage patching, primarily steel plates and composite fiber-reinforced materials [10,11]. The reinforcement methods are mainly divided into three: external bonded reinforcement, external prestressing reinforcement, and near-surface mounted reinforcement. The external prestressing reinforcement involves using CFRP bars [12] or steel bars [13] for achieving reinforcement outside the structure. This method is difficult to implement and is limited by the space between the bridge deck slabs. The near-surface mounted reinforcement involves embedding the reinforcement materials into the original structure for reinforcement [14]. This method damages the original structure when implemented. The comparison shows that the external bonded reinforcement is the most ideal. The application of steel plates in a concrete bridge deck slab reinforcement is somewhat limited because they are prone to corrosion and have poor durability [15,16,17]. In contrast, composite fiber-reinforced materials are corrosion-resistant, have high specific strength, and are fatigue-resistant. Currently, many scholars are conducting research on external carbon-fiber-reinforced polymers (CFRP) reinforcement technology. Mhanna et al. [18] conducted an experimental study on the shear load capacity of reinforced concrete beams strengthened with CFRP laminates. Al-Saawani et al. [19] conducted an experimental study on FRP U-wrap anchorage. Al-Huthaifi et al. [20] conducted an experimental study on reinforced concrete beams with longitudinal circular holes, using the CFRP strips strengthening method. Toutanji et al. [21] conducted a fatigue test study on concrete beams reinforced with CFRP. Al-Ghrery et al. [22] explored the effect of surface concavity of concrete structures on the performance of adhered CFRP reinforcement. Li [23], Yang [24], and Yang [25] collated, summarized, and analyzed the reinforcement technology of FRP in concrete structures. Zhou [26] concluded from an experimental study that the damage modes of CFRP-strengthened reinforced concrete beams under fatigue loading are mainly fatigue crushing of concrete in the compression zone, fatigue fracture of steel, and fatigue peeling at the interface. Research on U-shaped jacket [27], anchorage [28], and near-surface mounted reinforcement techniques [29] has advanced and developed to address the fatigue peeling damage of CFRP. In addition, CFRP is used for non-conventional reinforcement, such as the use of CFRP ropes for reinforcing RC joints under seismic loading [30]. CFRP reinforcement in concrete structures has been tried in various ways and the technology has matured. Jinping [31] repaired and strengthened the deck slab of an existing bridge by increasing the deck slab thickness and the amount of reinforcing steel, which improved the bearing capacity of the deck slab. This reinforcement method increases the self-weight of the structure, but its cost is large. External CFRP reinforcement is non-destructive to the structure and has a low cost. Therefore, reinforcement methods such as external CFRP cloth and CFRP mesh have certain advantages for concrete bridge deck slab reinforcement.

The fatigue damage process of reinforced concrete ribbed girder bridge deck slabs is complex, making it difficult to determine a reasonable and effective reinforcement method to extend their service lives. At present, research on deck slab strengthening technology is far less than that on other components, such as beams and columns [32], and studies on fatigue-strengthening technology of reinforced concrete ribbed girder bridge deck slabs are even fewer. Therefore, it is of practical significance to develop an external laminated steel plate and CFRP material to strengthen a bridge deck slab and study the changes in its fatigue life.

To facilitate inspection and maintenance, the external steel plate reinforcement can be cut into strips, pasted at a certain distance from the bottom of the plate, and anchored at the end [33,34,35,36]. Liqiang et al. [37] conducted a fatigue test study on steel-UHPC bridge deck slabs reinforced with steel plate strips. Xiaosan et al. [38] carried out a computational analysis of reinforced concrete slabs strengthened by compression steel plates. Qing et al. [39] carried out the theoretical derivation by reinforcing the original structure with a combination of steel plate and concrete. An experimental study of the strip-steel plate reinforcement of reinforced concrete (RC) ribbed girder bridge deck slabs has not yet been conducted. The strip-shaped steel plate reinforcement refers to the deck plate reinforcement method of the Shadun Port Bridge in China, with a steel plate size of 1150 mm × 200 mm × 10 mm, net distance between the strips of 200 mm, and anchor bolt size of M12 × 150 mm.

Oh et al. [40,41] used CFRP cloth to strengthen bridge deck slabs and concluded that the life of bridge deck slabs was significantly extended after strengthening. Drar et al. [42] performed a fatigue analysis on FRP-strengthened reinforced concrete slabs under moving loads. Teng et al. [43] conducted structural performance analysis on FRP-strengthened reinforced concrete slabs under cyclic loading of moving wheels. Shill et al. [44] conducted a flexural test study on a two-way concrete slab reinforced with basalt, and Martin Noël et al. [45] studied the shear performance of the post-tensioned FRP reinforced concrete slabs, under static and fatigue loading. In general, few studies have been conducted on CFRP-cloth-reinforced RC ribbed girder bridge deck slabs. Referring to literature [7,46] on the CFRP cloth-reinforcement method, the width of the cloth strips was selected as 150–200 mm, and the net distance between the strips was selected as 200–300 mm for the reinforcement of RC ribbed girder bridge deck slabs. At present, there are no reports related to CFRP mesh in the reinforcement of RC ribbed girder bridge deck slabs. Referring to the literature [8,47], CFN200/200 bidirectional CFRP mesh was selected to strengthen the deck slab of an RC ribbed girder bridge in this study.

This study focused on the fatigue damage of concrete ribbed girder bridge deck slabs by performing fixed-point equal-amplitude fatigue loading tests with reduced-scale test girders to explore the complementary effect of commonly used strengthening methods on the fatigue resistance of RC ribbed girder bridge deck slabs and the ability to restore and extend their service life. The novelty of the research reported in this paper is that it explores suitable strengthening methods for the fatigue resistance of reinforced concrete ribbed girder bridge deck slabs. The research addresses the safe operation of the deck slabs of reinforced concrete ribbed girder bridges in service and provides guidance on the engineering application, which will be carried out in depth in real bridge strengthening in the future.

## 2. Materials and Methods

### 2.1. Experimental Materials

The concrete selected was C40 small stone self-compacting concrete with a ratio of m_cement_:m_water_:m_sand_:m_crushed stone_:m_slag powder_:m_additives_ = 1:0.4:1.5:1.34:0.48:0.04. The measured 28 d concrete compressive strength was 42.1 MPa, and the elastic modulus was 3.23 × 10^4^ MPa.

HRB400 and HPB300 steel bars were used as reinforcements. The measured yield strengths were 415 MPa and 322 MPa, and the moduli of elasticity were 2.03 × 10^5^ MPa and 2.08 × 10^5^ MPa, respectively.

The CFRP cloth used consisted of unidirectional fabric and epoxy resin glue. The density of the CFRP cloth was 200 g/m^2^, theoretical thickness was 0.111 mm, tensile strength was 3550 MPa, and the elastic modulus was 2.4 × 10^5^ MPa. The CFRP-cloth-impregnated resin index is shown in Table 1, and the impregnated resin adhesive met the requirements of Class A adhesives.

The CFRP mesh consisted of a CFN200/200 bidirectional mesh and epoxy resin glue. The width of the carbon fibers was 5 mm, spacing was 20 mm, and the elastic modulus was 2.4 × 10^5^ MPa. The surface density of the CFRP mesh was 80 g/m^2^, and the theoretical thickness was 0.044 mm. The tensile strength was 4300 MPa.

The steel plate was Q235 steel with a thickness of 2.5 mm, connected by anchor bolts, and anchored by steel plate beading. A modified epoxy resin was used as the adhesive, and the index values are listed in Table 2. The adhesive met the requirements of Class A adhesives.

### 2.2. Samples

**Figure 1:**
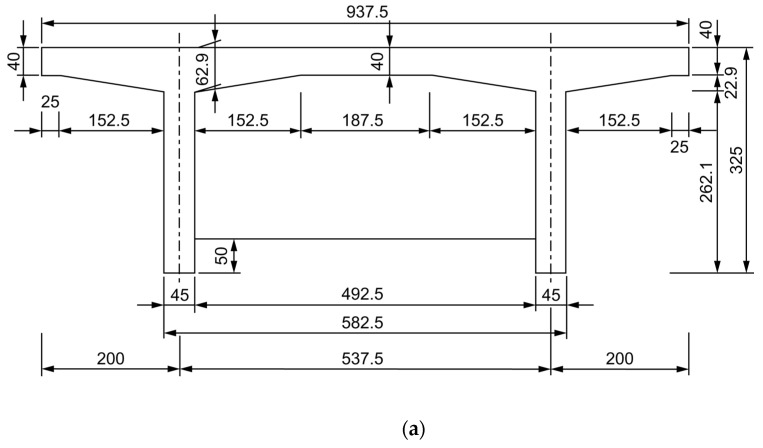

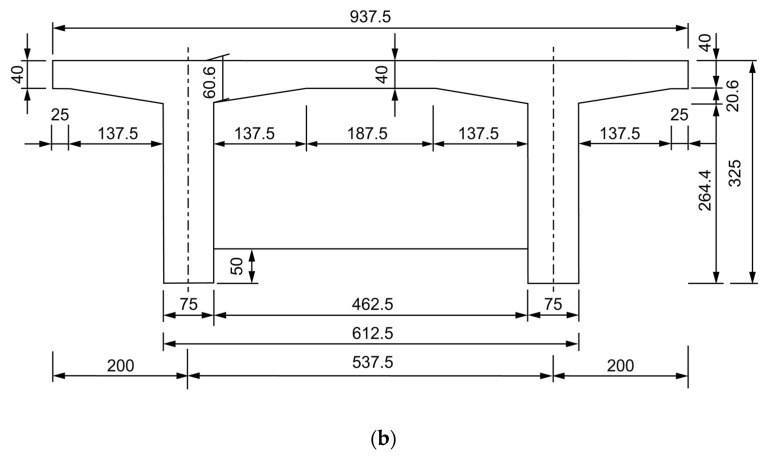
Cross-sectional arrangement of the test beam (mm) (**a**) Test beam midspan section size, and (**b**) Cross-sectional arrangement of the test beam support points.

**Figure 2:**
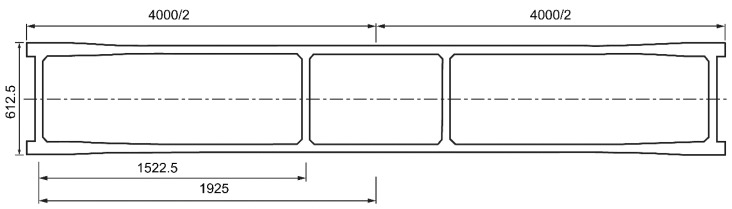
Top view of the test beam.

When designing the geometry of the tested beam, the size of the operating space and loading capacity of the laboratory fatigue tester should be taken into consideration. The length of the beam is 4 m, and the bottom dimension at the end of the beam is appropriately thickened and reinforced with more steel. The test beam was designed based on the standard drawing of the 16-m T-beam issued by the Ministry of Transportation in China, with a 1:4 scale reduction. The calculated span of the test beam was 3.85 m, and the two T-beams, wet joints, and transverse partitions were simultaneously cast as one. The concrete grade used was C40. The cross-sectional arrangement of the test beam is shown in Figure 1, and the top view is shown in Figure 2. The test beam was designed with two mid-transverse partitions. A one-way slab with an aspect ratio of 2.8 was considered as the focus of the study.

**Figure 3:**
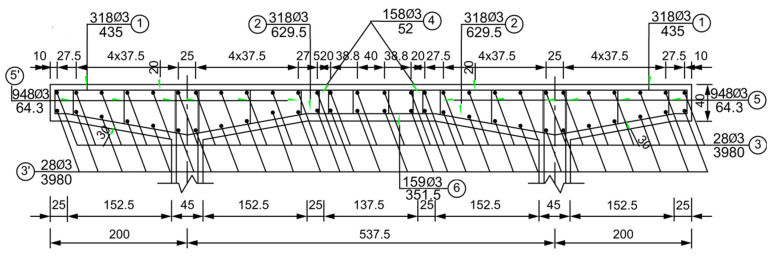
Bridge deck slab reinforcement.

The reinforcement of the test beam was the same as that of the standard drawing 16-m T-beam. For an equal reinforcement rate, the internal reinforcement of the test girders was 1/4 of the internal reinforcement diameter of the 16-m T-girders, thus ensuring the similarity of the structure. The test beam deck plate reinforcement is shown in Figure 3 (The numbers in the circle indicate the steel number). Five test beams were fabricated, three of which were reinforced; the specific working conditions are listed in Table 3. After the test beams were fabricated, they were maintained for 28 d for beam reinforcement treatment and testing.

Three test beams were selected and reinforced with CFRP cloth, CFRP mesh, or strip steel plates. The reinforcement method is external paste type, to paste one layer of reinforcement material. Since the one-way slab is stressed on the short side, CFRP sheets and steel plates are only reinforced in the cross-bridge direction. According to the 1:4 scaling requirements, the width of the CFRP cloth was 50 mm and the clear distance between the fabric strips was 50 mm; cross-bridge reinforcement and a 100-mm-wide compression bar were set at the end of the fabric strips. The CFRP mesh was directly reinforced because it was difficult to scale the design down. The reinforcement area percentages of the bridge deck slab with the CFRP sheet, CFRP grid, and strip steel plate are 50%, 100%, and 50%, respectively. The reinforcement area percentages with the CFRP sheet and strip steel are the same.

**Figure 4:**
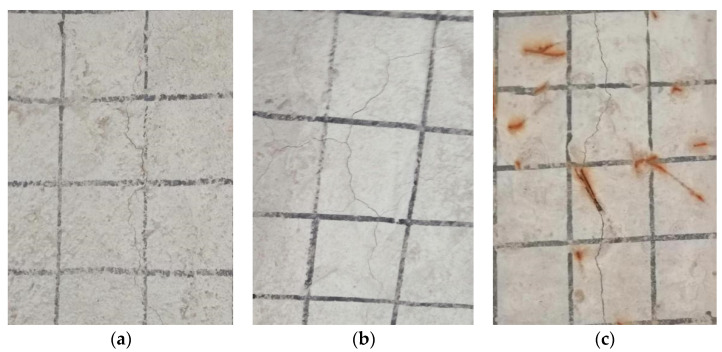
Signs of Lattice Crack in Bridge Deck under Fatigue Loading (**a**) PL3, (**b**) PL4, and (**c**) PL5.

**Figure 5:**
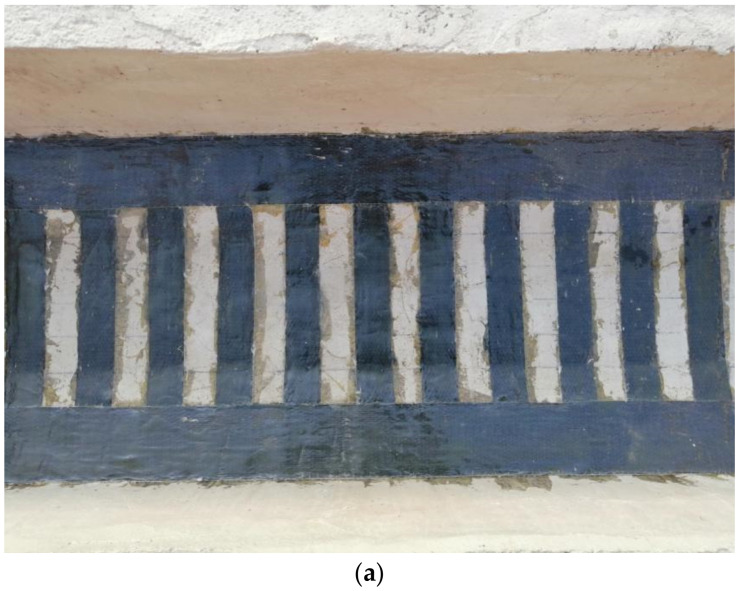

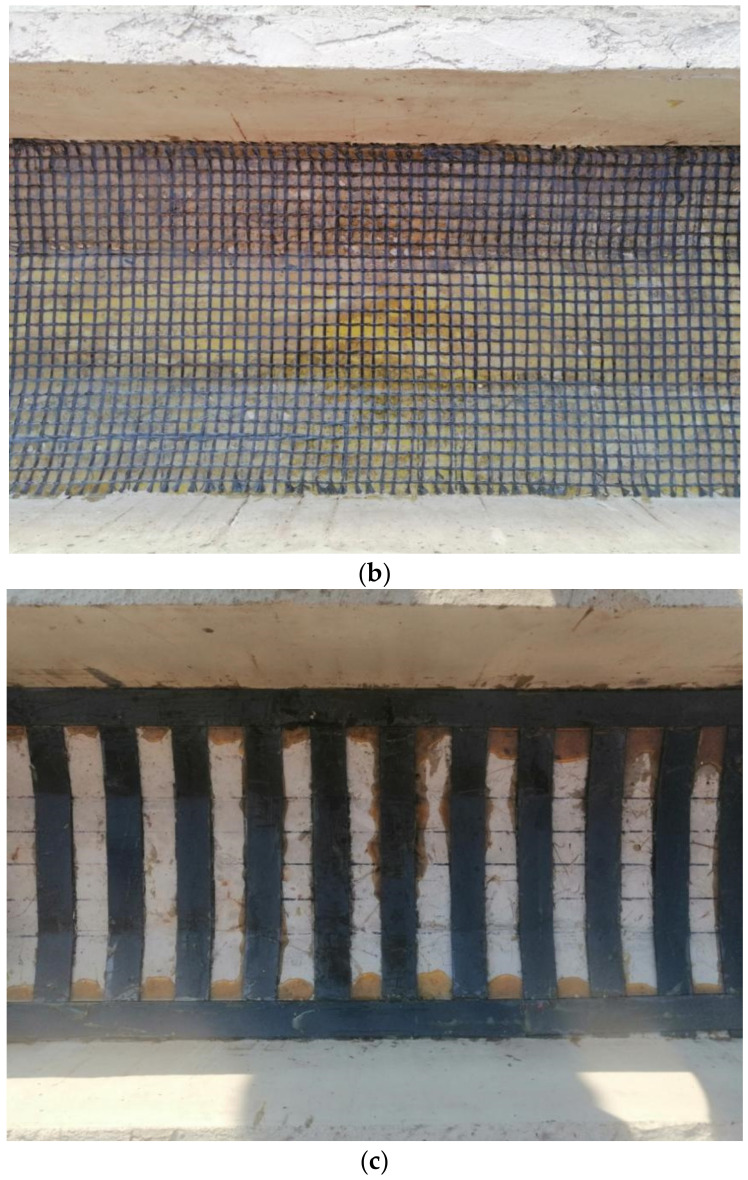
Reinforced test beam (**a**) CFRP cloth reinforcement, (**b**) CFRP mesh reinforcement, and (**c**) Strip plate reinforcement.

Before reinforcement, the test beam was fatigue loaded. The fatigue load level was set to 0.515. The loading point was selected as the middle of the one-way slab near the end of the beam, and the loading area was 0.1 m × 0.2 m. When signs of lattice cracks appeared under the deck slab and the width of the main crack reached 0.1 mm, the fatigue loading was stopped; at this time, the signs of cracks appeared in the test beam, as shown in Figure 4. Then, the test beam was removed from the loading device, and the deck slab was reinforced. The reinforced test girders are shown in Figure 5.

### 2.3. Test Setup, Instrumentation, and Loading Procedures

**Figure 6:**
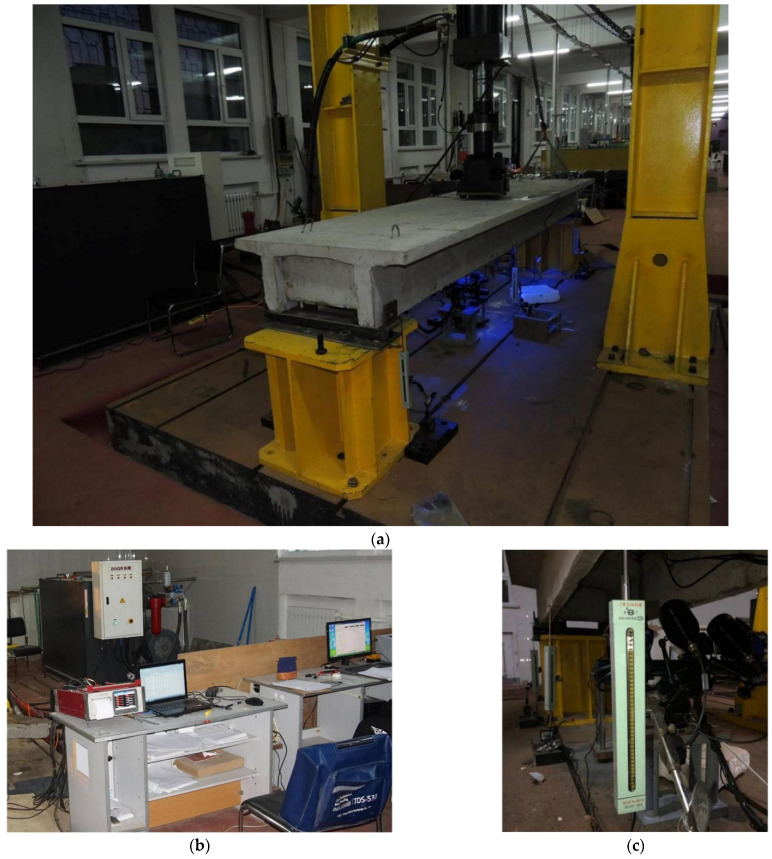
Fatigue test loading of test beam (**a**) Loading device, (**b**) Control System, and (**c**) Displacement test systems.

A servo actuator was used to simulate the wheel load loading. The loading area was 0.1 m × 0.2 m, and fine sand was placed under the loading plate. A PLS-500 electro-hydraulic servo dynamic and static test system was used for the static load and fatigue tests. The system consists of a servo linear actuator, a constant pressure servo pump station, an oil distribution module, a full digital servo controller, a computer data processing system, a loading frame and attachments, etc. The electro-hydraulic servo-controlled actuator is used to load the tested beam. The fatigue test was set to constant-amplitude fatigue, and the loading is shown in Figure 6. The test process is carried out in a closed-loop control.

**Figure 7:**
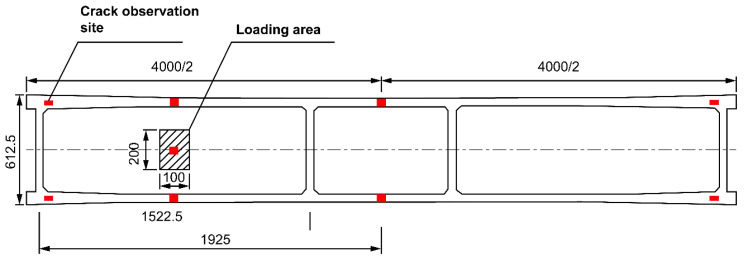
Loading position and displacement gauge field layout (mm).

In addition to using concrete strain gauges, the concrete strain values during fatigue were collected using a TDS-530 high-speed static data acquisition instrument. The YHD-200 model was selected as the displacement sensor. A crack observer and ARAMIS professional non-contact measurement systems were used to observe the cracks. The site layout is shown in Figure 7.

Formal testing began after pre-loading was completed, and the system was normal. A static load test was performed using monotonic graded loading. Each level of loading is generally 1/10 of the ultimate load. For the fatigue test, the minimum value of the load was 1 kN, and the maximum value was 37.4 kN. The control method of the process spectrum is “force control”, loaded with a sinusoidal load wave. The loading frequency was set at 5 Hz. The predetermined numbers of fatigue cycles were: 2, 5, 10, 50, 100, 150, and 200, and the unit was “ten thousand times”. After the numbers of fatigue cycles were reached, the test machine automatically stopped, and a static load test with F_max_ as the loading force was performed. The fatigue damage criteria were fatigue fracture of reinforcing steel or a positive section crack width of 1.5 mm.

## 3. Results and Discussion

### 3.1. Failure Modes and Crack Development Patterns

**Figure 8:**
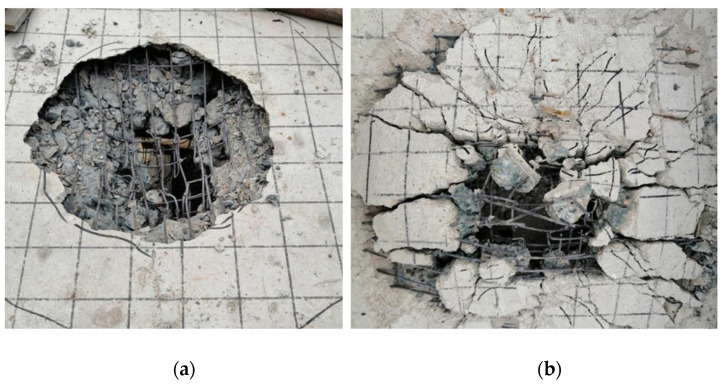
Fatigue test failure of PL1 test beam (**a**) Damage to upper part of bridge deck and (**b**) Damage to lower part of bridge deck.

**Figure 9:**
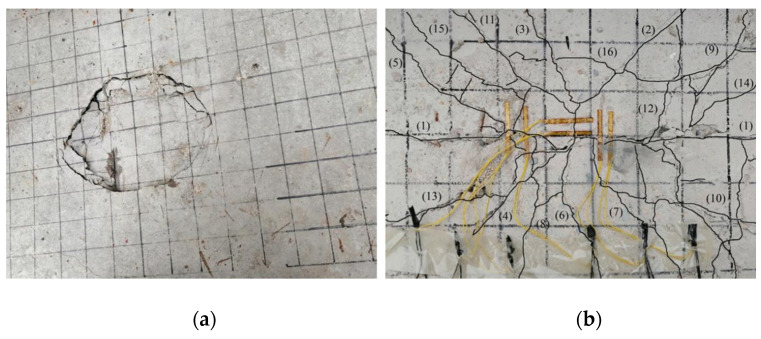
Fatigue test failure of PL2 test beam (**a**) Damage to upper part of bridge deck and (**b**) Damage to lower part of bridge deck.

**Figure 10:**
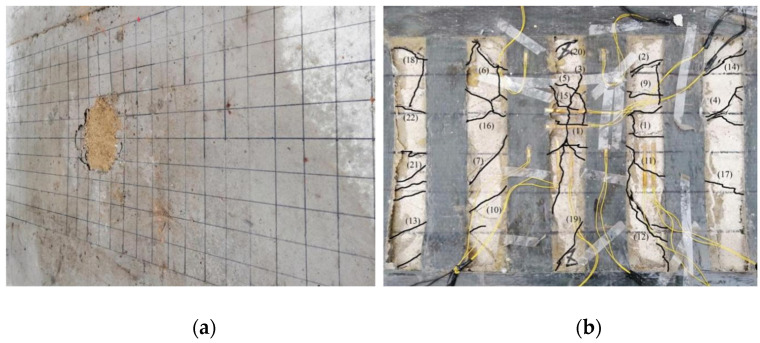
Fatigue test failure of PL3 test beam (**a**) Damage to upper part of bridge deck and (**b**) Damage to lower part of bridge deck.

**Figure 11:**
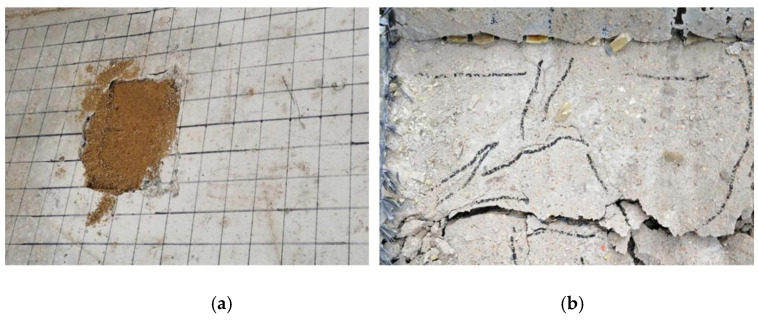
Fatigue test failure of PL4 test beam (**a**) Damage to upper part of bridge deck and (**b**) Damage to lower part of bridge deck.

**Figure 12:**
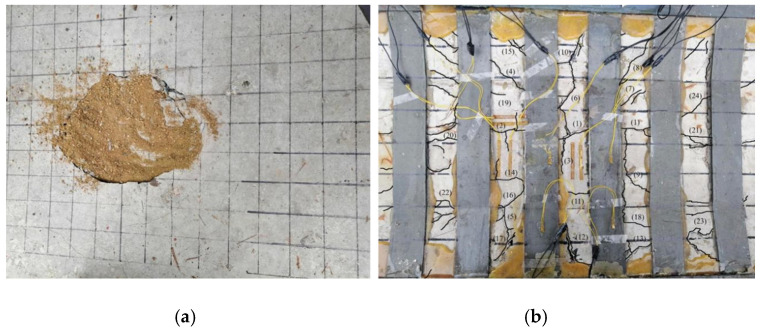
Fatigue test failure of PL5 test beam (**a**) Damage to upper part of bridge deck and (**b**) Damage to lower part of bridge deck.

A static load damage test was performed on the PL1 test beam with a cracking load of 31.45 kN and ultimate load of 74.23 kN. Punching shear damage occurred at the loading point of the bridge deck slab, as seen in Figure 8. The minimum value of the reciprocating load of the PL2–PL5 test girders was 1 kN, and the maximum value was 37.4 kN. The maximum value was because of the PL1 ultimate load and fatigue load level of 0.515. The PL2 deck slab exhibited a fatigue fracture at the main crack. At the moment of fracture, the concrete in the compressed area at the loading point was crushed, and punching shear damage occurred, as shown in Figure 9. During the fatigue test, all reinforced deck slabs exceeded two million fatigue cycles. The fatigue life was approximately 1.6 to 2 times that of an unreinforced beam. The reinforced deck slab was fatigue-fractured at the main crack, and a fracture sound could sometimes be heard at the time of fracture. At the moment of fracture, the concrete in the pressure zone at the loading point was crushed, and punching shear fatigue damage occurred. As the PL4 cracks were difficult to observe, the CFRP mesh was broken and lifted for observation after the test. The fatigue damages of PL3, PL4, and PL5 are shown in Figure 10, Figure 11 and Figure 12. The fatigue damage of the reinforced deck slab appeared as brittle damage. The area around the hole was similar to that of the fatigue damage of the unreinforced deck slab, and it was smaller than the area around the hole in the static load damage. Regarding the fatigue failure characteristics, the fatigue failure of reinforced bridge deck slabs is mainly in the form of brittle fracture of reinforced steel. In fatigue failure, the reinforcing material peels. Finally, under the shear of the load, the steel will be pulled out by fatigue, and the bridge deck will collapse and lose its bearing capacity. Overall, after reinforcement, the bridge deck slab improved its ability to resist dynamic loads, reduced its brittleness, and slightly improved its ductility.

In the fatigue test, the crack development in the reinforced bridge deck slabs was relatively slow. The PL2 deck slab cracks were divided into four stages. In the initial development stage, fine cracks developed mainly in one direction. In the stable development stage, transverse and longitudinal cracks with lattice-like signs were observed. In the rapid development stage, cracks with a seam width of 0.2 mm or more propagated rapidly, and new cracks appeared between the existing cracks. In the damage stage, the longitudinal cracks instantly became the primary damage cracks, and the crack width reached 0.45 mm. The cracks were roughly distributed radially, with lattice-like signs in the center, and the number and length of crack roots were greater than those in the static load test.

PL3, PL4, and PL5 were subjected to fatigue loading before reinforcement approximately 500,000 to 750,000 times. Reinforcement was performed when cracks with lattice signs appeared. The fatigue crack development processes of PL3 and PL5 bridge deck slabs were similar. During the initial development stage, cracks with lattice signs developed to some extent with the increase in fatigue loading cycles; however, because of the restraining effect of the reinforcement material, lattice-like cracks tended to propagate slowly. During the stable development stage, transverse, longitudinal, and lattice cracks formed. In the rapid development stage, cracks with a width of more than 0.2 mm rapidly increased, and new cracks appeared between the existing cracks. At this point, the concrete and reinforcement material interfaces began to separate. In the damage stage, transverse or oblique cracks quickly became the main cracks of destruction. The crack width reached 0.4–0.5 mm, and the cracks were roughly radially distributed during damage. There were lattice-like signs in the center, and the number and length of crack roots were greater than those of unreinforced PL2. The PL4 deck slab, because of the two-way restraint effect of the CFRP mesh, had a lower crack expansion speed than those of PL3 and PL5. The number and length of crack roots were smaller than those of PL3 and PL5 during the damage stage. There were no radial cracks, and the punching and cutting cracks at the junction of the deck slab and beam rib were the main damage cracks. The experiments showed that the tensile effect of the reinforcement material restrained the development of longitudinal deck slab cracking.

The fatigue cracks that developed in the PL2, PL3, and PL5 deck slabs are summarized in Table 4. The cracks in PL4 are not included in Table 4 because they were not observable. The number of cracks in the reinforced deck slabs at the stable development stage was the same as that in the unreinforced deck slab. The number of major cracks in the rapid development stage was six to eight (37.5–50%) more than that in the unreinforced deck slab. This indicates that the reinforced deck slabs have a greater density of cracks, and the reinforcement material limits the development of major cracks in the stable development stage. The slow propagation of the main cracks leads to many new cracks in the bridge deck in the rapid development stage, and the crack density increases accordingly. The slow propagation of the main cracks substantially prolongs the service life of the bridge deck.

### 3.2. Concrete and Reinforcement Strain Variation Patterns

**Figure 13:**
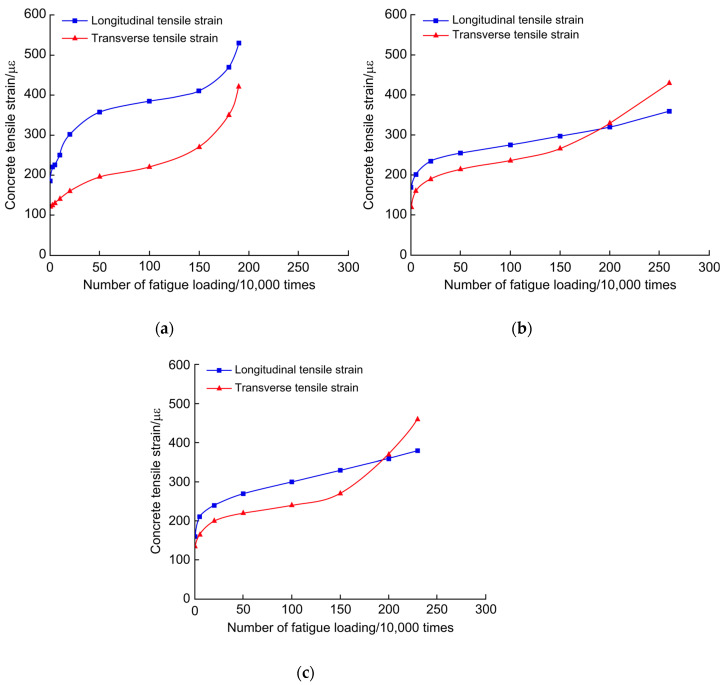
Change in concrete tensile strain under the slab based on number of fatigue loading cycles (**a**) PL2, (**b**) PL3, and (**c**) PL5.

The data obtained from the measurement and observation system were combined and plotted against the change in the concrete tensile strain under the test beam slab with the number of fatigue cycles, as shown in Figure 13. PL4 is missing in Figure 13 because the observation system could not directly observe the PL4 concrete tensile strain. As shown in Figure 13, the concrete tensile strain exhibited linear growth with plastic accumulation damage during the early stage of fatigue. The concrete plastic deformation under the slab slowed down at the middle stage of fatigue, and at the late stage of fatigue, the concrete tensile strain increased rapidly, while the damage continued to increase. The transverse tensile strain of the PL2 bridge deck slab was always larger than the longitudinal tensile strain, and the transverse tensile strain became the main tensile strain for fatigue damage in the late fatigue stage. The transverse tensile strains of the PL3 and PL5 bridge deck slabs developed slowly with the restraining effect of the reinforcement material. In comparison, the longitudinal tensile strain developed more rapidly than the transverse tensile strain and finally became the main tensile strain of the fatigue damage. From the reinforcement materials, the performance of the CFRP cloth in inhibiting the cumulative fatigue deformation of the tensile strain was better than that of the strip steel plate. Regarding the growth rate, the growth rate of the concrete transverse tensile strain of reinforced deck slabs is significantly lower than that of unreinforced deck slabs. As shown in Figure 13b,c, at the late stage of degradation, the degradation rate of unreinforced concrete in the longitudinal direction of the bridge deck slab is greater than that of transverse concrete. This indicates that in one-way slab reinforcement, two-way reinforcement is more beneficial for extending the remaining life of the bridge deck slab.

**Figure 14:**
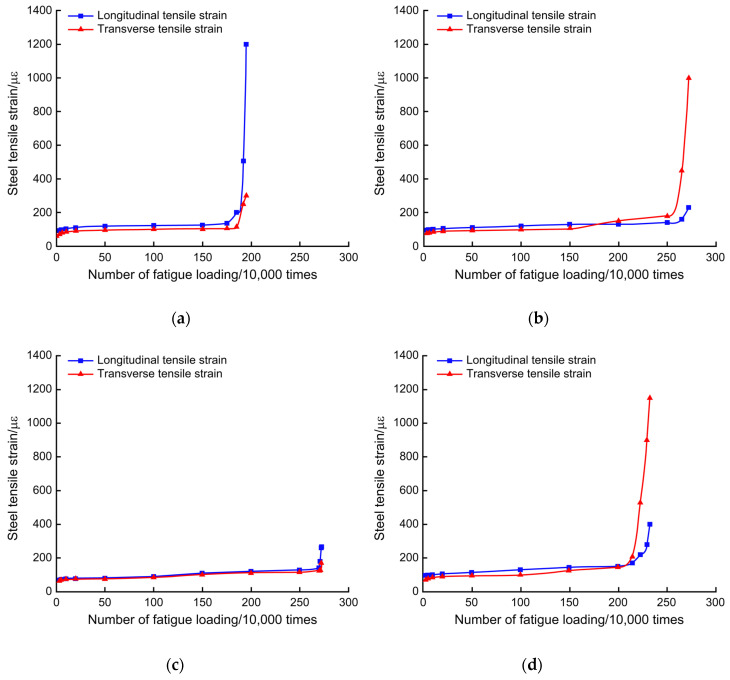
Change in steel tensile strain under the slab based on number of fatigue loading cycles (**a**) PL2, (**b**) PL3, (**c**) PL4, and (**d**) PL5.

Figure 14 shows the variation in the tensile strain of the reinforcement in the test beam deck slab under fatigue loading. As shown in Figure 14, the transverse tensile strain of PL2 was closer to the longitudinal tensile strain, and its value was lower than those of PL3 and PL5. In the middle and early stages, the longitudinal tensile strain was less than the transverse tensile strain; in the later stage, the longitudinal tensile strain exceeded the transverse tensile strain. Finally, the longitudinal tensile strain significantly increased. The transverse tensile strain of the PL4 bridge deck was relatively close to the longitudinal tensile strain, and its value was lower than those of PL3 and PL5. When the fatigue cycle is 1 million times, the incremental value of the transverse tensile strain of reinforcing steel in the reinforced deck is approximately 50–75% of that of the unreinforced deck, and that of the longitudinal tensile strain of reinforcement is approximately 40% to 50%. In terms of growth rate, the degradation process of reinforcement steel in reinforced deck slabs is slower than in unreinforced deck slabs. These test results indicate that the CFRP mesh can strengthen the bridge deck in both directions, and the fatigue damage of the steel bars develops slowly after the bridge deck is reinforced.

### 3.3. Deflection Development and Fatigue Degradation Patterns

**Figure 15:**
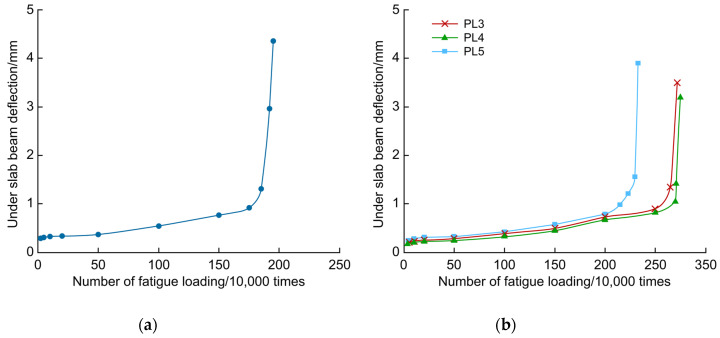
Relative curves between beam deflection and fatigue cycles under the slab at loading point (**a**) Unreinforced beam and (**b**) Reinforced beam.

Figure 15 shows the changes in the deflection under the PL2–PL5 bridge deck slabs with fatigue loading. The deflection developed faster at the beginning of the test and near the fatigue damage. In the middle of the test, the deflection developed smoothly. The deck plate deflection was the largest when the fatigue life was reached. In the middle of the test, the growth rate of the reinforced deck plate deflection was smaller than that of the unreinforced deck plate, and the deflection of the reinforced deck plate was slightly smaller than that of the unreinforced deck plate for the same number of fatigue cycles. The deck plate reinforced with strip steel plates was the first to experience stiffness degradation, earlier than CFRP-cloth- and CFRP-mesh-reinforced deck slabs by approximately 500,000 cycles. The curve of deflection change in this test is identical to the curve shape reported in the literature [7], both of which are in the late fatigue stage, and the bridge deck slabs are rapidly damaged.

The degree of deterioration *D* is an index characterizing the deterioration condition of the bridge deck plate, which differs from the linear cumulative fatigue damage criterion and is calculated according to the following equation:

(1) $$D=\frac{\delta -\delta_{0}}{\delta_{c}-\delta_{0}},$$

where 0≤D≤1; δ is the measured value of deflection under a slab under a live load; $\delta_{0}$ is the theoretical deflection value of the full section under a live load, taken from the deflection value at a static load to the upper limit of fatigue loading before the fatigue test; $\delta_{c}$ is the theoretical deflection value of the cracked section under a live load, taken from l/600.

**Figure 16:**
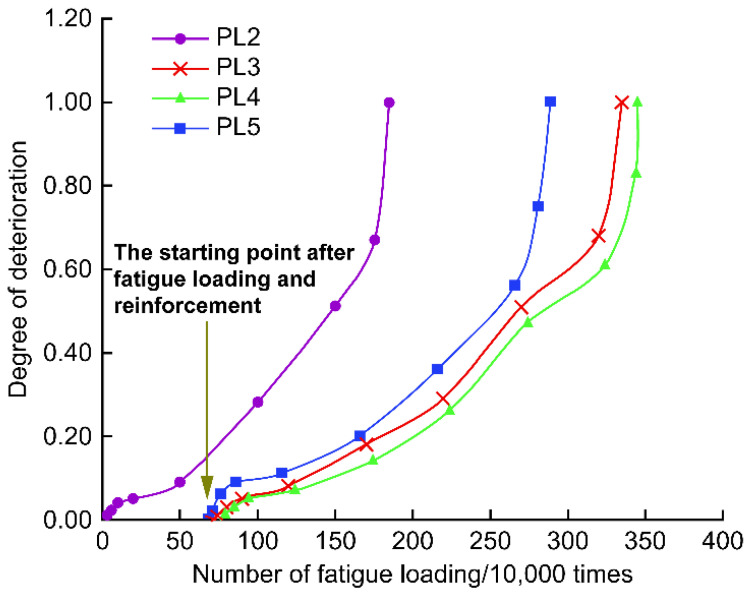
Comparison of deterioration degree.

Using Equation (1), the fatigue test deflection data were calculated for the degradation degree *D*, and the *D–N* relationship curve was plotted, as shown in Figure 16. The degradation is not linear but non-linear cumulative fatigue damage. The degradation degree *D* develops with the number of fatigue *N* in three phases. The initial phase is a slow deterioration phase, with a deterioration degree *D* value of less than 0.1, accounting for approximately 20% of the life. The late degradation was similar for the reinforced and unreinforced decks. In the middle stage, the deterioration rate of the reinforced deck plate decreased significantly. From fatigue cycles 500,000 to 1.8 million, the *D* of the unreinforced plate increased from 0.1 to 0.7. From 1.2 million to 2.8 million fatigue cycles, the *D* of the strip-steel plate reinforcement developed from 0.1 to 0.7. From 1.2 million to 3.2 million fatigue cycles, the *D* of the CFRP cloth reinforcement developed from less than 0.1 to 0.7. Therefore, the deterioration rate of the reinforced deck was significantly slower than that of the unreinforced deck, and considering the reinforcement material, the CFRP cloth was better than the strip steel plate at limiting the deterioration rate.

The deterioration value $D_{i}$ is an indicator of the degree of deterioration of the bridge deck at each moment and is calculated according to the following formula:

(2) $$D_{i}=\frac{\delta_{i}-\delta_{i-1}}{\delta_{c}-\delta_{0}}$$

where $\delta_{i}$ is the measured deflection under a live load after the fatigue load cycle; $\delta_{i-1}$ is the measured deflection under a live load after the (*i* − 1)th fatigue load cycle.

**Figure 17:**
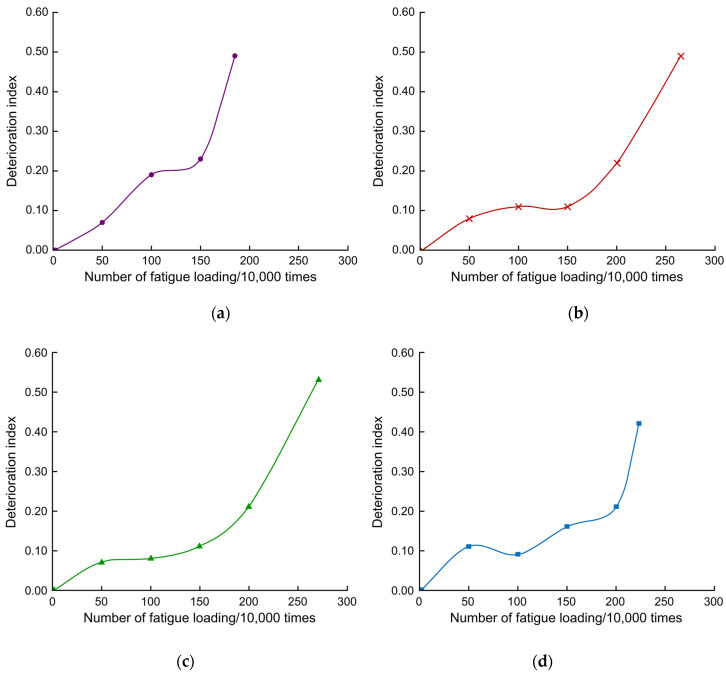
*D_i_–N* curves (**a**) PL2, (**b**) PL3, (**c**) PL4, and (**d**) PL5.

Using Equation (2), the fatigue test deflection data were subjected to the deterioration value $D_{i}$ calculation, and the $D_{i}$–*N* relationship curve was plotted, as shown in Figure 17. $D_{i}$ did not vary linearly with the number of fatigue cycles. At the beginning of fatigue, $D_{i}$ developed linearly with small values. In the middle of fatigue, $D_{i}$ did not change significantly, and at the end of fatigue, $D_{i}$ increased dramatically. At 1 million fatigue cycles, $D_{i}$ of the unreinforced deck plate was approximately 0.2, nearly 2 times larger than the reinforced bridge deck slab. $D_{i}$ of the CFRP cloth and steel plate strip reinforced deck plates were approximately 0.1, and $D_{i}$ of the CFRP mesh reinforced deck plate was less than 0.1. This indicates that reinforcement material can reduce the deterioration value of the deck plate by approximately 50% during the fatigue progression period, and the CFRP cloth is better than the strip steel reinforcement at suppressing the deterioration value of the deck slab.

The stiffness reduction factor $\theta_{f}$ is an index characterizing the deterioration of the bridge deck plate stiffness with fatigue development and is calculated according to the following equation:

(3) $$\theta_{f}=\delta_{0}/\delta_{f},$$

where $\theta_{f}$ is related to the load level S and number of load cycles N; $\delta_{f}$ is the deflection deformation value of the load action after the reciprocal load action, then the static load test, corresponding to the upper limit of the reciprocal load.

**Figure 18:**
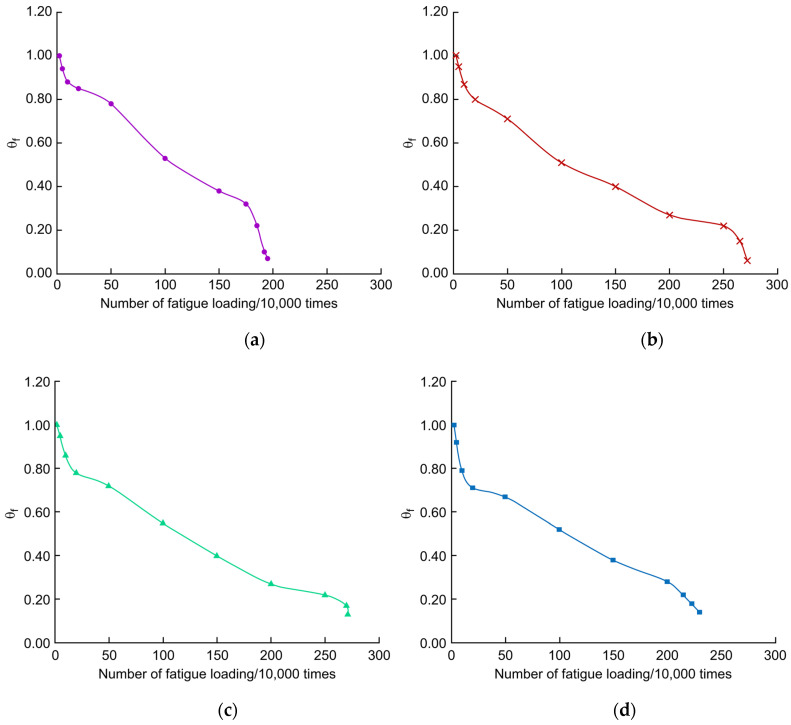
$\theta_{f}$–*N* curves (**a**) PL2, (**b**) PL3, (**c**) PL4, and (**d**) PL5.

Using Equation (3), $\theta_{f}$ was calculated for PL2–PL5 under fatigue loading, and the $\theta_{f}$–*N* relationship curve was plotted, as shown in Figure 18. $\theta_{f}$ generally decreased linearly with the number of fatigue cycles. The $\theta_{f}$ of the unreinforced deck decreased faster than those of the reinforced decks. At 1 million fatigue cycles, the $\theta_{f}$ for each unreinforced and reinforced deck was around 0.5. This indicates that the stiffness degradation process is similar in the early stages of fatigue loading. When the fatigue loading was at 2 million cycles, the unreinforced deck $\theta_{f}$ was approximately 0.05, and the reinforced deck $\theta_{f}$ was approximately 0.3. This indicates that in the middle and later stages of fatigue loading, that is, after the appearance of fatigue cracks, the stiffness of the bridge deck decreases slowly, and the reinforcement material reduces the stiffness deterioration of the bridge deck, thereby extending its remaining life.

### 3.4. Effect of Reinforcement on the Service Life of Bridge Decks

**Figure 19:**
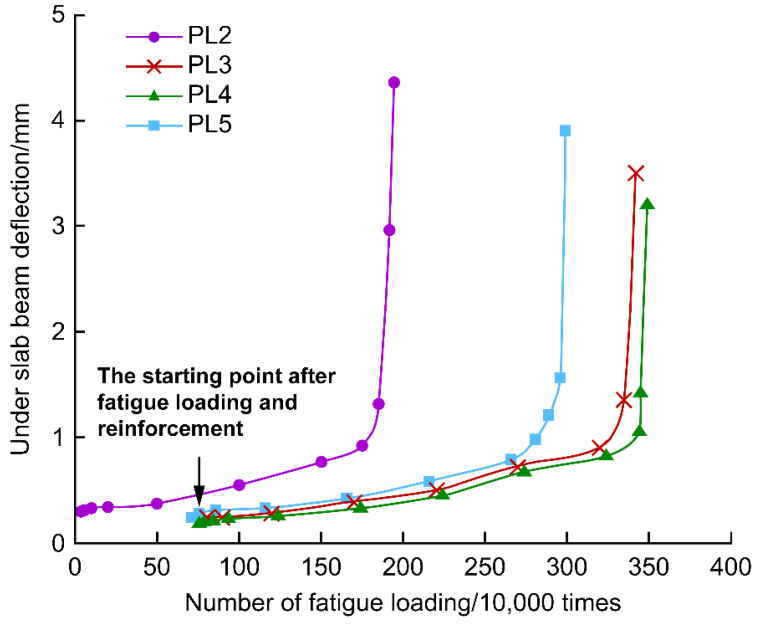
Relative deflection under the slab at loading point of test beam.

The comparative life analysis of fatigue reinforcement based on the under-slab deflection test data is shown in Figure 19 and plotted against the number of fatigue cycles before reinforcement for the PL3–PL5 test beams.

Compared with PL2, the late fatigue inflection points and final damage numbers increased by 82.9% and 75.4% for PL3, 85.1% and 78.9% for PL4, and 52% and 53.3% for PL5, respectively. The CFRP cloth and CFRP mesh significantly improved the fatigue resistance of the deck plate, and the strip steel plates showed some improvement in the fatigue resistance of the bridge deck slabs. The CFRP cloth and CFRP mesh lasted 22.1–25.6% longer than the bridge deck slabs reinforced with strip steel plates. In general, after fatigue damage occurred in the bridge deck plate, if the deck plate was reasonably reinforced, the fatigue life of the bridge deck plate was extended, and the reinforcement effect of the CFRP cloth and CFRP mesh was better than that of the strip steel plate under the same fatigue load.

The cumulative damage degree *D* of the bridge deck slab is based on the linear cumulative fatigue damage criterion, and is calculated according to the following equation:

*D = N_D_/N_f_,* (4)

where *N_D_* is the cumulative number of fatigue cycles performed, and *N_f_* is the cumulative number of fatigue cycles when the fatigue damage is reached.

Previous research [48] provides the relationship between the deterioration process and cumulative damage degree. *D* was 0–0.2 during the latent period, 0.2–0.5 during the progressive period, 0.5–0.8 during the accelerated period, and 0.8 to 1.0 during the deterioration period. From the perspective of prevention and preservation, Japan’s longlife repair plan states that the reinforcement period after the bridge deck is damaged must be between the advanced and accelerated periods. A previous study [7] determined that the reinforcement period of the PL3–PL5 bridge decks after damage is approximately at the end of the progress period (*D* = 0.41), which is similar to the longlife repair concept of the Japanese bridge deck and conforms to the prevention and preservation strategy. From the test data of PL3–PL5, after the bridge deck is reinforced once, the service life of the bridge deck is extended by approximately 53.3–78.9%, indicating that the reinforcement has a significant influence on the service life of the bridge deck.

In terms of reinforcement materials, lightweight, high-strength, and corrosion-resistant CFRP materials have better fatigue reinforcement prospects than strip steel. Therefore, CFRP cloth strip reinforcement is more suitable for the fatigue reinforcement of bridge deck slabs.

### 3.5. Discussion of the S-N Curve of Bridge Deck Slabs under Fatigue Reinforcement

Wang [49] established a fatigue life prediction model for reinforced concrete ribbed girder bridge deck slabs based on fatigue damage product theory and test data under fatigue loading of concrete deck slabs with wheel movement, and the resulting *S-N* curves were as follows:

*Log* (*F_i_*/*F_u_*) = −0.0557*LogN* − 0.1737 (5)

where, *F_i_* is the base load value (KN), *Fu* is the ultimate punching shear load capacity of the concrete deck slab (KN), and N is the number of cycles.

Combining the fatigue test data in this paper and the punching and cutting theory of the bridge deck slab, the *S-N* curve of the fatigue strengthened reinforced concrete ribbed girder bridge deck slab is obtained on the basis of Equation (5), as shown in Equation (6):

*Log* (*F_i_*/*F_u,c_*) = −0.0361*LogN* − 0.1037 (6)

where *Fu,c* is the impact shear- bearing capacity of the bridge deck plate after reinforcement.

In the literature [7], the fatigue performance of reinforced concrete bridge deck slabs with FRP material reinforcement was studied, and the *S-N* curve of reinforced concrete slabs was established, as shown in Equation (7):

*Log* (*P*/*P_SX.c_*) = −0.06417*LogN* + *Log*0.850 (7)

where *P_SX.C_* is the punch-cut load capacity of the reinforced concrete deck slab reinforced by FRP.

Comparing Equations (6) and (7), it can be concluded that the two *S-N* curves have the same form and the parameters do not differ much; however, the slope of the *S-N* curve of Equation (6) is smaller, and the curve is relatively flat. Since a steel–concrete composite girder deck slab was the target in the literature [7], this indicates that the *S-N* curves of the reinforced deck slab will be different depending on the type of deck slab.

## 4. Conclusions

In this study, deck slabs of a reinforced concrete ribbed girder bridge with a reduced dimensional design were strengthened, and fatigue tests were conducted, which led to the following conclusions.

1. Under fixed-point equal-amplitude fatigue loading, reinforced deck slabs produced radial cracks and punching shear damage.
2. When the number of load cycles exceeded 90% of the fatigue life, fatigue main cracks appeared in the reinforced deck slab at the loading point.
3. The fatigue life of bridge deck slabs was approximately 53.3–78.9% longer, when they are reinforced at the accumulated damage degree of 0.4. The fatigue life of bridge deck slabs reinforced with CFRP cloth or CFRP mesh was 22.1–25.6% more than that of bridge deck slabs reinforced with strip steel plates.
4. Under the same fatigue load, both CFRP cloth and CFRP mesh were more effective than the strip-steel plate reinforcement for the fatigue reinforcement of bridge deck slabs. From a health monitoring perspective, CFRP cloth is more suitable for the fatigue reinforcement of bridge deck slabs.

This study, based on experimental data, explores the feasibility of strengthening bridge deck slabs with CFRP and other materials. In the future, finite element modeling and analytical modeling of prefabricated specimens will be carried out to obtain the fatigue damage law under different reinforcement conditions.

## Figures and Tables

**Table 1:** Performance index of carbon fiber impregnated in resin.

Density g/cm^3^	Tensile Strength MPa	Compressive Strength MPa	Tensile Shear Strength MPa	Flexural Strength MPa	Tensile Bond Strength MPa	Modulus of Elasticity MPa	Elongation %
1.1–1.3	40.1	78.6	19.4	66.9	3.6	2500	2.4

**Table 2:** Safety performance index of adhesive for sticking steel plate.

Tensile Strength MPa	Compressive Strength MPa	Flexural Strength MPa	Positive Tensile Bond Strength to Concrete MPa	Steel–Steel Bond Tensile Strength MPa	Tensile Modulus of Elasticity MPa	Elongation %
32.5	70.5	55.4	3.4	36	3500	1.8

**Table 3:** Test beam conditions.

Test Beam Number	Test Beam Condition	Loading Method	Loading Instructions
PL1	Baseline test girders with no reinforcement of the bridge deck slab	Bridge deck slab static load damage test	Continuously load until the deck slab is damaged, and determine the ultimate bearing capacity of the deck slab P_u,TL_
PL2	Deck slab without reinforcement, fatigue loaded to deck slab damage	Bridge deck plate isometric fatigue test	F_min_ = 1 kN, F_max_ = 0.515 P_u,TL_
PL3	Fatigue reinforcement with CFRP cloth, fatigue loaded to deck slab failure		
PL4	Fatigue reinforcement using CFRP mesh, fatigue loaded to deck slab failure		
PL5	Fatigue reinforcement using strip steel plates, fatigue loaded to deck plate failure		

**Table 4:** Fatigue crack development in reinforced beams.

Specimen Number	Upper Fatigue Limit (kN)	Number of Major Cracks during the Stable Development Stage	Number of Major Cracks during the Rapid Development Stage (Late)	Maximum Width of Cracks during the Damage Stage (mm)
PL2	37.4	5	16	0.45
PL3	37.4	4	22	0.42
PL5	37.4	5	24	0.48

## Data Availability

The data presented in this study are available on request from the corresponding author.
